# Regulatory hurdles for genome editing: process- vs. product-based approaches in different regulatory contexts

**DOI:** 10.1007/s00299-016-1990-2

**Published:** 2016-05-03

**Authors:** Thorben Sprink, Dennis Eriksson, Joachim Schiemann, Frank Hartung

**Affiliations:** Institute for Biosafety in Plant Biotechnology, Julius Kuehn Institute, Erwin-Baur-Str. 27, 06484 Quedlinburg, Germany; Department of Plant Breeding, Swedish University of Agricultural Sciences, Sundsvägen 10, 23053 Alnarp, Sweden

**Keywords:** New plant breeding techniques, Genome editing, Genetic engineering, Regulation, Site directed nucleases, CRISPR/Cas9, ODM

## Abstract

Novel plant genome editing techniques call for an updated legislation regulating the use of plants produced by genetic engineering or genome editing, especially in the European Union. Established more than 25 years ago and based on a clear distinction between transgenic and conventionally bred plants, the current EU Directives fail to accommodate the new continuum between genetic engineering and conventional breeding. Despite the fact that the Directive 2001/18/EC contains both process- and product-related terms, it is commonly interpreted as a strictly process-based legislation. In view of several new emerging techniques which are closer to the conventional breeding than common genetic engineering, we argue that it should be actually interpreted more in relation to the resulting product. A legal guidance on how to define plants produced by exploring novel genome editing techniques in relation to the decade-old legislation is urgently needed, as private companies and public researchers are waiting impatiently with products and projects in the pipeline. We here outline the process in the EU to develop a legislation that properly matches the scientific progress. As the process is facing several hurdles, we also compare with existing frameworks in other countries and discuss ideas for an alternative regulatory system.

## Introduction

The development of plant breeding methods in modern times is always preceded by scientific progress. Gregor Mendel’s laws of genetic inheritance launched an era of controlled hybridizations and selection of superior crop material, and further discoveries in biology, physics and chemistry paved the way for the astonishing yield increases in all major crops particularly in the latter half of the twentieth century. Discoveries in molecular biology enabled more targeted approaches and expanded the available gene pool in plant breeding in the 1980s, allowing researchers and breeders to work efficiently at the single-gene level with the genetic material from any organism. The regulatory system put in place in the European Union (EU) in 1990 (The Council of the European Communities 1990a) focused mainly on the distinction between conventional plant breeding techniques involving hybridizations and induced mutations on one hand and recombinant DNA technology involving DNA from sexually non-compatible species on the other. This was at that time a relatively clear distinction, with the term “genetically modified organism”, or GMO, coined to represent the latter category. However, scientific progress in the last two decades has moved plant breeding into a new continuum between genetic engineering and the so-called conventional methods. With novel genome editing (GE) techniques, the focus shifts from the insertion of protein-encoding DNA towards the use of regulatory RNA molecules and/or site-specific DNA-modifying enzymes.

A battle is currently raging in the EU on how to accommodate novel GE techniques within the existing regulatory frame for GMO. The discussions have intensified in 2014 and 2015 with precedential cases both from the industry and from public researchers. The current regulatory system in the EU is both process- and product-oriented; however, it has by and large been interpreted as strictly process-based. It is along this interpretation that the main battle line is now drawn, with the novel GE techniques ending up on either side of the GMO definition. In this review, we outline the political steps taken towards the regulation of novel GE techniques in the EU and put this in relation to other regulatory contexts such as that of Canada, the US and Argentina. We also make the case for an interpretation including both process and product, with focus on the latter, and discuss suggestions for an alternative regulatory system in the EU that may improve the current legislation regarding the incorporation of science-based, neutral and experience-based decision making.

## Crop genetic improvement technologies

Plant breeding for the improvement of plant-derived products used for human nutrition, feeding of domesticated animals or raw material production has been performed for thousands of years. Crossing of superior plants obtained by selection breeding has been, for a long time, the only possible method to improve cultured plants. Traditional breeding techniques have been complemented, since the last century by conventional mutagenesis, translocation breeding and intergeneric crosses leading to a more sophisticated exploitation of the existing natural genetic variation. With the development of genetic engineering in the 1980s, plant breeding made a movement from cisgenic to transgenic approaches resulting in transgenic plants in which genes from non-crossable organisms are introduced by different transformation techniques. The transgenic plants are produced by undirected approaches integrating the transgene (or cisgene) in unspecified locations of the genome. Since then the development of breeding techniques progressed rapidly resulting in much more sophisticated methods to create plants with novel traits. These techniques are summarized as New Plant Breeding Techniques (NPBT, Lusser et al. 2011). In particular, the genome editing and modification techniques described in the following are tools for sequence-specific changes in the plant genome. These techniques enable breeders to introduce a single point mutation or a new DNA sequence at a specific location in the plant genome, thereby circumventing the negative side effects of conventional mutagenesis. The potential risks of exploring these new genome editing techniques are comparable to conventional mutagenesis or transgene technology (EFSA 2012). Considering these techniques and emerging new breeding techniques, the GMO-legislation framework in the EU, which is mainly interpreted and executed as being based on the technique which is used to produce a new plant, is not reflecting the progress made in recent development of NPBT.

In 2013, the European Academies Science Advisory Council (EASAC) has provided a comprehensive report on the risks and benefits of crop genetic improvement technologies, a term which is including NPBTs, genetic engineering and emerging plant breeding techniques. The report did not find evidence for an intrinsic higher risk of genetic engineering in comparison to conventional breeding technologies. This finding is based on solid science conducted in several thousand research projects and published in the last 20 years. The EASAC report came to the conclusions that “the trait and product, not the technology, in agriculture should be regulated, and the regulatory framework should be evidence-based” (EASAC 2013). This request for a trait-/product-based regulation reflects the scientific evidence which is very solidly based on GMO safety research and risk analyses accumulated in the last two decades (Heap 2013; Swiss National Science Foundation 2012; Hartung and Schiemann 2014). The EASAC report was endorsed by several academic organizations, most prominently by Anne Glover, former Chief Scientific Adviser to the President of the European Commission (EC). “The conclusions of the report are based on the best possible evidence and I endorse its conclusions whole-heartedly.” Besides the EASAC statement mentioned above that intrinsic risks of genetic engineering do not exist, concerning the NPBTs Ann Glover stated that “*…* we shouldn’t forget that there are also other promising novel plant breeding technologies, post-GM, and we shouldn’t make the mistake of regulating them to death as we have done with GM” (Glover 2013).

In its recent Statement on New Breeding Techniques (EASAC 2015), EASAC requests that the EU policy development for agricultural innovation should be transparent, proportionate and fully informed by the advancing scientific evidence and experience worldwide. EASAC demands to resolve current legislative uncertainties and asks EU regulators to confirm that the products of NPBTs, when they do not contain foreign DNA, do not fall within the scope of GMO legislation. In contrast, in an Open Letter to the Commission on new genetic engineering methods, the anti-GMO Non-Governmental Organizations (NGOs) call on the Commission to reject any attempt to exclude these new techniques from EU regulation (NGO 2015). In particular, they urge the Commission to ensure that organisms produced by these new techniques will be regulated as GMOs under existing EU regulations and that current GMO health and environmental safety testing requirements are strengthened in light of the enhanced ability of these new techniques to alter the genetic code.

## GMO regulatory frame in the European Union

**Fig. 1:**
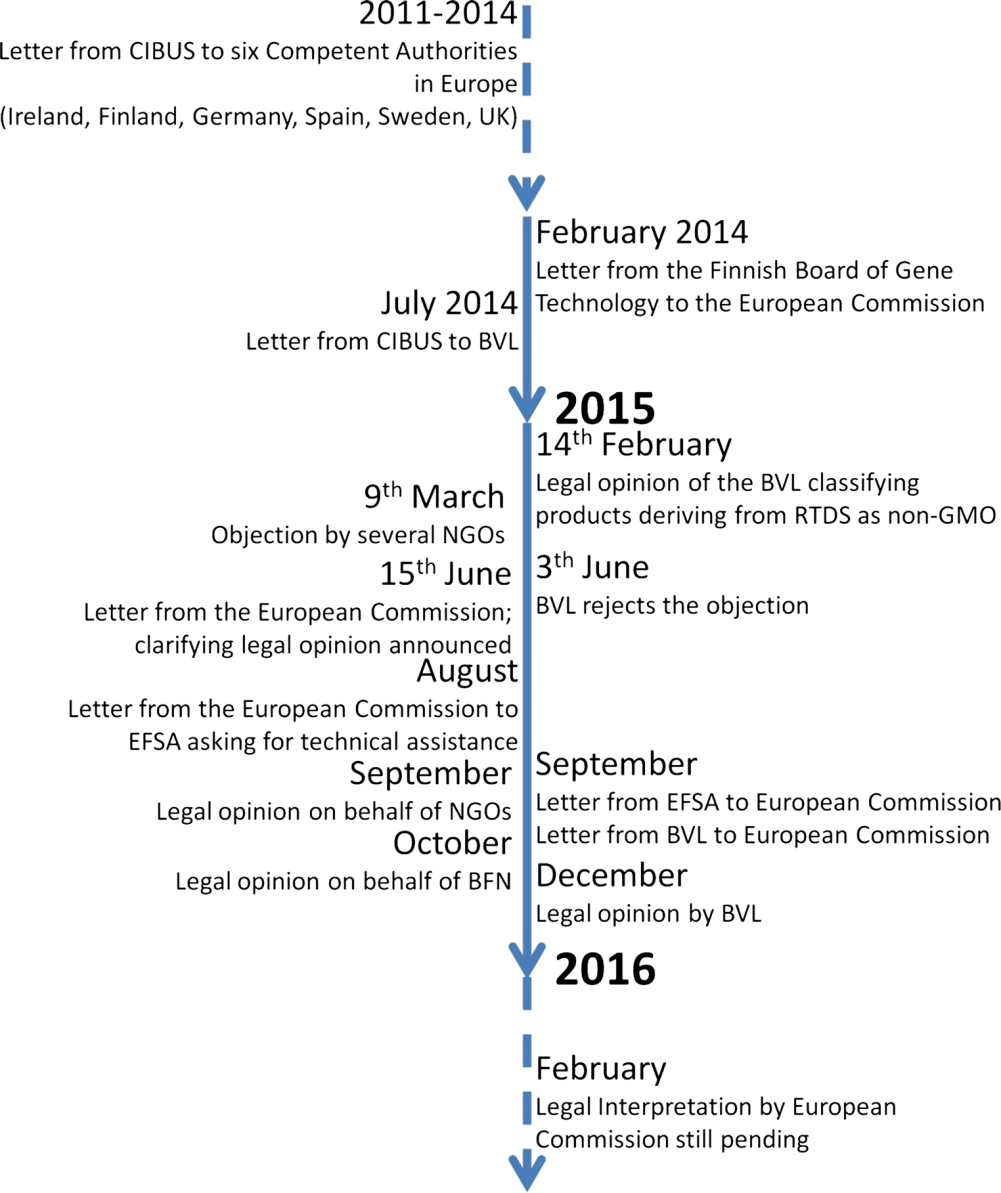
Timeline of the debate on the legal interpretation of genome editing techniques and resulting crops in the European Union

The NPBTs are very heterogeneous and might or might not involve steps in which a genetic modification (genetic modification in the context of this manuscript means that a recombinant DNA sequence has been introduced) of the plant genome occurs. However, the resulting plant or parts of it like fruits often does not possess a genetic modification. According to the EU definition which is included in the first EC Directives on the contained use of genetically modified micro-organisms (90/219/EC) and deliberate release in the environment of genetically modified organisms (90/220/EC) established in 1990, a GMO is “an organism in which the genetic material has been altered in a way that does not occur naturally by mating and/or natural recombination” (The Council of the European communities 1990a, b). This definition is in line with the Cartagena Protocol on Biosafety to the Convention on Biological Diversity adopted on 29 January 2000 and entered into force on 11 September 2003 (European Parliament and European Council 2003). The EU signed the Protocol on 24 May 2000 and ratified it on 27 August 2002. The EU Directives have been revised several times, resulting in the actual Directives 2001/18/EC and 2009/41/EC (European Parliament and European Council 2001, 2009). Both Directives list techniques that (1) give rise to genetic modification (Annex I, Part A of Directive 2009/41/EC and Annex IA Part 1 of Directive 2001/18/EC); (2) are not considered to result in genetic modification (Annex I, Part B of Directive 2009/41/EC and Annex IA Part 2 of Directive 2001/18/EC); (3) yield organisms that are excluded from the Directive (Annex II Part A of Directive 2009/41/EC and Annex IB of Directive 2001/18/EC). These Annexes still originate from 1990 and consequently do not match the development of modern breeding techniques. Due to this, the legal discussion concerning NPBTs accelerated in the last two years as the first plants generated with NPBTs have been requested for release. In 2014, the Finnish Competent Authority asked the EC for assistance to a request from the company CIBUS© (Finnish Ministry of Social Affairs and Health and Board for Gene Technology 2014). Already, in 2011, the company started to request opinions on a herbicide-tolerant oilseed rape line, created with the ODM technique RTDS™ (rapid trait development system), from six Competent Authorities in Europe including the German Federal Agency for Consumer Protection and Food Safety (BVL) (European Biotechnology 2015). The UK DEFRA and the Swedish Gene Technology Advisory Board followed by the other Competent Authorities including the BVL expressed their opinion that plants developed by RTDS™ should not be regarded as GMO in relation to the Directives 2001/18/EC and 2009/41/EC, as the RTDS™ technology is a mutagenesis approach which is not involving recombinant nucleic acids (BVL 2015a; Collected correspondence from Competent Authorities 2014–2015). The Competent Authorities additionally stated that, if by any means the EC comes to a different evaluation, this opinion has to be reconsidered. The official notification from the BVL, which was based on a statement by the German Biosafety Commission (ZKBS; Zentrale Kommission für Biologische Sicherheit), was released on 5 February 2015. Shortly after, on 9 March, an association of several NGOs claimed an objection (For a complete timeline see Fig. 1). The NGOs association defined the oilseed rape created by RTDS™ as a GMO, based on the definition in the Directive 2001/18/EC (Brockmann 2015). The BVL repudiated after extensive examination of the objection on 3 June, confirming its previous notification (BVL 2015b). On 15 June, the EC informed the competent authorities of all member states that, until the legal status of NPBTs would be clarified, a protective approach should be implemented (European Commission 2015). In addition, the EC announced a clarifying legal analysis to be released by end of 2015 and requested technical assistance from EFSA (European Food Safety Authority) in August 2015 related to the legal analysis of NPBTs. In its answer, the EFSA stated that ODM techniques as well as SDN-1 and -2 at present are used to create point mutations only. These mutations are identical to those introduced via natural or induced mutagenesis and thus can be considered as a form of mutagenesis. If due to technological advancement, this definition is not applicable anymore, further analysis may be needed (EFSA GMO unit 2015). This statement is consistent with the view of the new technology working group (NTWG) and gives no legal classification on plants created using these approaches (Lusser et al. 2011).

In the same letter, EFSA regarded RdDM as a type of epigenetic regulation that can impact gene expression without altering the nucleotide sequence of the DNA. Following this definition, the term alteration of the genetic material is not applicable for RdDM (EFSA GMO unit 2015). In addition to EFSA, BVL sent a letter to the EC in September 2015 with the request to forward it to the legal service to help clarifying the legal status of NPBTs. The BVL had combined the views of UK, Irish and German Competent Authorities leading to the assessment that Directive 2001/18/EC is both product- as well as process-oriented (BVL 2015c).

Several NGOs released a legal analysis in September 2015, in opposition to the BVL notification, claiming that the Directive 2001/18/EC was misinterpreted and that all NPBTs (including ODM) should fall under the scope of GMO regulation as the analysis comes to the interpretation that the European gene technology law is strictly process-based (Krämer 2015; Table 1). A second legal analysis on behalf of the German Federal Agency for Nature Conservation (BFN) assisted these opinions. Besides providing the same legal interpretation, it additionally includes RdDM as a genetic engineering technique (Spranger 2015; Table 1). Both legal analyses interpret the Directive 2001/18/EC as strictly process-based and state that all NPBTs make use of recombinant DNA and thus have to be regulated as GMOs, regardless of whether a stable transformation occurred or the ability of the nucleic acid to replicate in a living cell.

**Table 1:** Comparison of SDN-1, -2, and -3 in relation to the legal interpretations (BVL, NGOs, BFN, NTWG, ZKBS, EFSA)

	BVL^1^	ZKBS^2^	NTWG^3^	EFSA^4,5^	NGOs^6^	BFN^7^
SDN-1	Non GMO	Non GMO	Non GMO	Non GMO	GMO	GMO
SDN-2	Non GMO	Non GMO	Non GMO	Non GMO	GMO	GMO
SDN-3	GMO	GMO	GMO	GMO^b^	GMO	GMO
ODM	Non GMO^a^	Non GMO	Non GMO	Non GMO	GMO	GMO
RdDM	n.d	Non GMO	Non GMO	Non GMO	n.d	GMO
Interpretation	Process/product	n.d	n.d	n.d	Process	Process

The classification refers to plants generated by using these techniques without stable integration of recombinant DNA

*SDN* site-directed nucleases, *ODM* oligonucleotide-directed mutagenesis, *RdDM* RNA-dependent DNA methylation, *n.d* no opinion given, *GMO* genetically modified organism, *BVL* German Federal Agency for Consumer Protection and Food Safety, *ZKBS* Zentrale Komission für biologische Sicherheit, *NTWG* New technology working group, *EFSA* European Food Safety Authority. *1* BVL 2015d, *2* ZKBS 2012, *3* Lusser et al. 2011, *4* EFSA 2012, *5* EFSA GMO unit 2015, *6* Krämer 2015, *7* Spranger 2015

^a^Serial steps should be considered separately

^b^Due to the known target site of the transgene lesser amounts of event-specific data might be necessary for the risk assessment

On 7 December 2015, the BVL released a legal opinion maintaining its assessment decision from 5 February 2015 (BVL 2015d). In this legal opinion, the BVL is interpreting the Directive 2001/18/EC as process- and product-based (Table 1). Furthermore, it is stated that oligonucleotides used in ODM approaches as well as guide-RNAs used in CRISPR approaches are not recombinant DNA, since they are not a novel combination of genetic material. Therefore, products created using SDN-1, -2 or ODM approaches should not be regarded as GMO according to the Directive 2001/18/EC. This is in accordance with the assessment of several scientific organizations in Europe, such as ZKBS and EFSA (EFSA 2012, 2015; ZKBS 2012). This interpretation reflects also the view of the NTWG (Lusser et al. 2011; Table 1).

In addition, national academies of science in Germany, such as the Leopoldina, acatech (German Academy of Science and Engineering), and the Union of the German Academies of Sciences and Humanities, the German Research Foundation (DFG) as well as the European Plant Science Organisation (EPSO) support the application of NPBTs for future crop improvement. An advantage to the classical mutation approaches is that only small and predefined loci are modified with many of these techniques (National academies of Sciences 2015; EPSO 2015). The academies also share the interpretation of BVL that the Directive 2001/18/EC should be interpreted as process- as well as product-based.

To date (April 2016), a clarifying legal opinion of the EC is still pending but might be released in the first quarter of 2016. Until the legal opinion is released the legal status of living organisms as well as products deriving from NPBT approaches is unclear.

## Legal interpretation of genome editing techniques

### Site-directed nucleases 1, -2 and -3

**Fig. 2:**
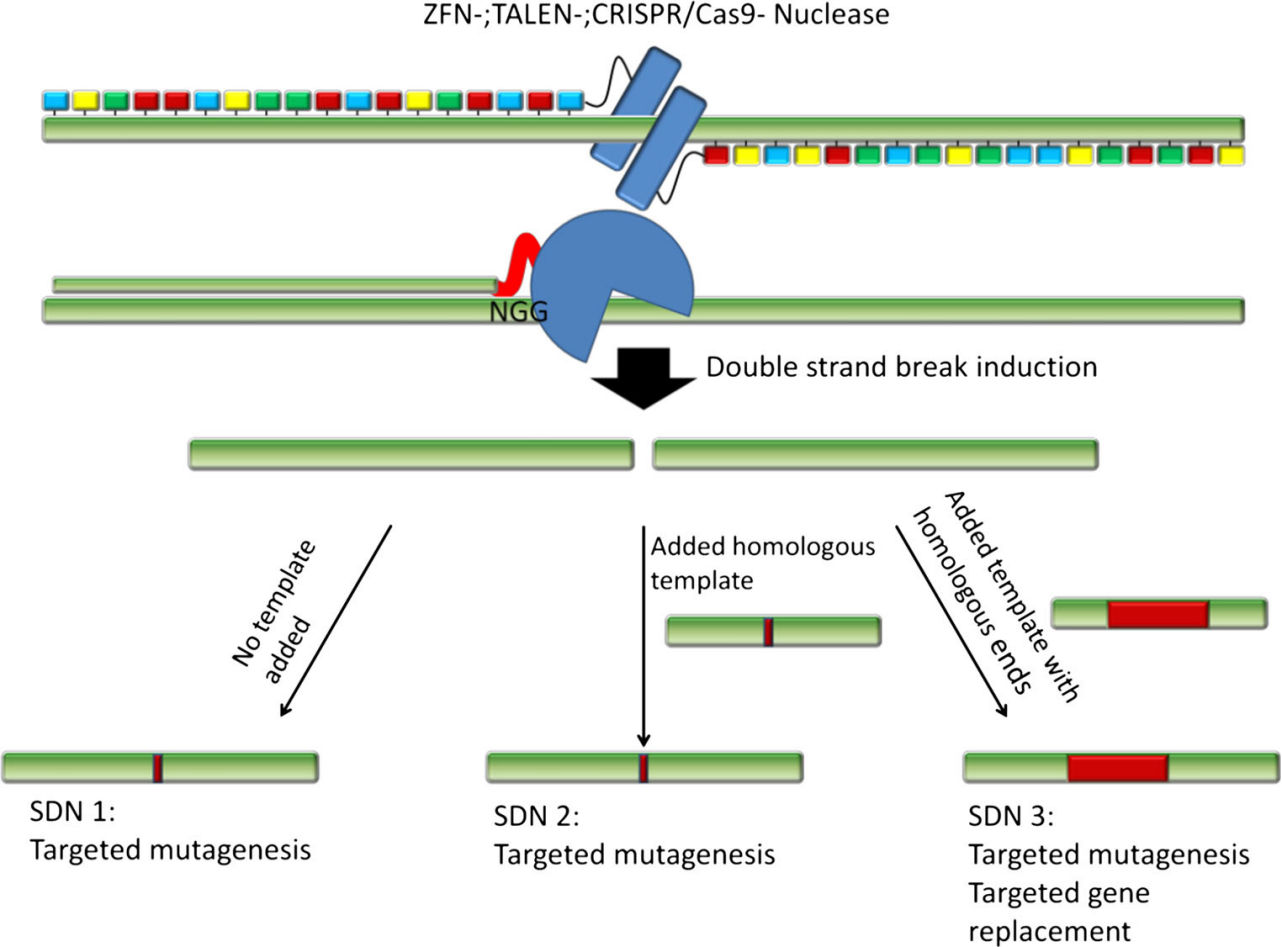
Overview of Site-Directed Nucleases techniques and the resulting genome editing. An SDN is introducing a double strand break which is the starting point for each gene editing approach. When this break is repaired via the host cellular repair mechanisms without the use of an added repair template, the approach is defined as SDN-1. When a homologous repair template is added and the break is repaired via HR using this template, the approach is defined as SDN-2. When the added template possesses DNA with homologous ends in combination with non-homologous sequences and the break is repaired via HR using this template, then recombinant DNA is added to the genome and the approach is defined as SDN-3

The opinions of the NTWG and the ZKBS concerning organisms resulting from SDN-1 and -2 approaches coincide. The resulting organisms carry mutations which originate from the cellular repair mechanisms non-homologous end joining and/or homologous recombination, both of which are natural DNA repair systems. These mutations are indistinguishable from natural or chemical-/radiation-based mutations not resulting in a GMO according to § 3 Nr. 3b. Satz 2 Buchst.a GenTG (Mutagenese) (GenTG 1993). Jones (2015) also clarifies that because of the cellular mechanisms involved, SDN can be considered as a mutagenic agent and as such being similar to the radiation or mutagenic chemicals used in classical mutation breeding. The difference is that changes induced by SDN-1 and -2 are intended to be site-specific. The ZKBS states that the added DNA used in SDN-2 approaches possesses only a few base pair (<20 bp) differences compared to the endogenous DNA; therefore, it is not considered as recombinant DNA (Fig. 2). SDN-3 approaches are considered differently, as plants arising from SDN-3 approaches carry a foreign DNA derived from added recombinant DNA. The resulting plants are considered as GMO in accordance with § 3 Nr. 3 GenTG. However, as the integration site of the DNA can be targeted, off-target effects are expected to be less compared to a classical transgenesis approach (Fig. 2). Mutations induced using SDN-1 and -2 cannot be traced back to the technique when no vector DNA remains in the modified organism. The changes created using SDN-3 can be traced back, and the resulting organism should fall under the scope of the GMO regulation (Lusser et al. 2011; ZKBS 2012). The legal analysis by the BVL agrees with this opinion, as it states that mutations induced by SDN-1 or -2 should not be rated as GMO in accordance with Directive 2001/18/EC: “Furthermore, the organisms are generated without the use of recombinant nucleic acids. Neither the oligonucleotides, as components of the mutagen in the ODM technique, nor the guide-RNAs used to apply the CRISP-Cas9 technique are recombinant nucleic acids in the sense of the Directive. Although the term “recombinant nucleic acids” is not defined in the Directive, the wording in Annex I A, Part 1, No. 1 implies that “recombinant nucleic acid techniques” must involve the formation of new combinations of genetic material. However, the oligonucleotides used in the ODM technique, with the exception of one or a few nucleotides, are identical to the corresponding site in the genome of the treated plant cells and, therefore, do not represent new combinations in the sense of new arrangements of genomic sequences.” In addition, the BVL interprets the Directive 2001/18/EC as both process- and product-based: “It cannot be derived from the wording of Article 2(2) that the GMO definition covers only the process by which the genetic modification is induced. Rather, it is also important that a product is created, whose genetic material has been altered in a way that would not be possible by the conventional breeding methods or natural processes. In this respect, the exclusion of natural events is directly related to the genetic material and not to the manner of modification. Referring to the phrase “in a way” as evidence of an exclusive reference to the process, on the other hand, is not convincing. This “way” is not necessarily to be translated in the sense of the way of production; instead the phrase “in a way” has also to be understood in its adjective form.” The BVL additionally states that this opinion is also aided by the Cartagena protocol: “The definition of the term GMO given in the Cartagena Protocol can also serve as an aid to interpretation. Article 3(g) of the Cartagena Protocol (international law) defines the term “living modified organism (LMO)” as “any living organism that possesses a novel combination of genetic material obtained through the use of modern biotechnology”. This definition clearly captures both the end product (living organism with a novel combination of genetic material) and the process (use of modern biotechnology).”

The legal analyses on behalf of the NGOs and BFN result in a different opinion, since the GMO regulation is strictly interpreted as process-based. As Krämer (2015) states: “It follows from the definition of GMO in Article 2(2) that Directive 2001/18 is a directive which is “process-based”: it covers organisms that are generated by a specific process (“the genetic material has been altered in a way..”). The Directive does not look at the final result of the process, the organism, but rather at the way in which this final result is obtained…. This means that Directive 2001/18 intends to regulate certain techniques which it considers of being able to constitute a risk to human health or the environment”. Spranger (2015) advance a similar view: “First of all, Annex I A Part 1 No. 1 essentially refers to the procedure of incorporation. From an applicability point of view, it is sufficient that an incorporation, as such is performed. The fact that this organism can in turn be reproduced without any further incorporation is irrelevant.” In addition: “it needs to be taken into account that Annex I A Part 1 No. 2 has to be interpreted in light of the aim of the European legislator who intended that the simple use of genetic modifying techniques would be sufficient for the applicability of Directive 2001/18/EC by the means of a process approach.”

### ODM

**Fig. 3:**
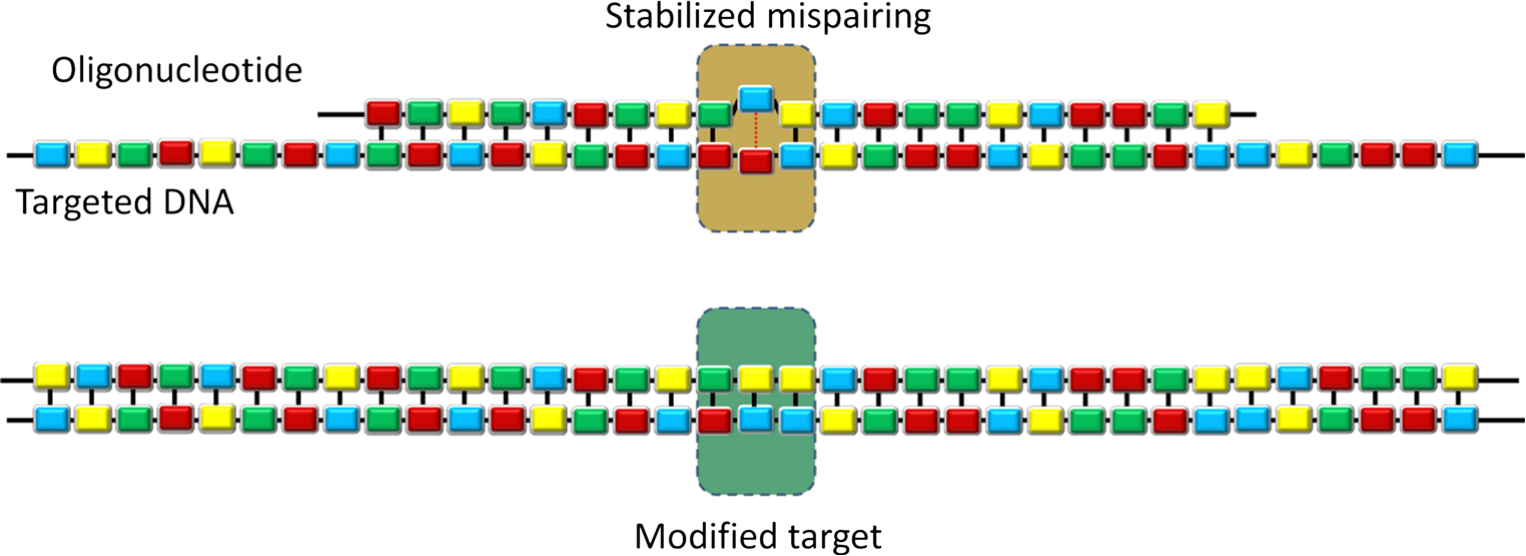
Overview on oligonucleotide-directed mutagenesis. A single stranded oligonucleotide with a modified single base is used to target a homologous DNA. The oligomer and the DNA form a stabilized mismatch. During DNA amplification, the mispaired nucleotide gets integrated in the complementary strand leading to a modified target sequence which is inheritable

The evaluation of organisms created using the ODM technique leads to coinciding opinions by the NTWG and the ZKBS. The oligonucleotides introduced into the cells are not a novel combination of genetic material, as the sequence of the oligomers is adjusted to the targeted sequence with one or a few changes (Fig. 3). In addition, the oligomers are neither recombinant DNA nor genetic material in accordance with § 3 Nr 3a Buchst. B GenTG (GenTG 1993). The oligomers act like a mutagenic substance which is introducing mutations ranging from one to a few base pairs which are indistinguishable from naturally occurring and chemical-/radiation-based mutations (Lusser et al. 2011; ZKBS 2012). The legal analysis by the BVL concludes with this opinion as mentioned above and additionally gives the following practical considerations which would make the regulation of plants generated in such a way practically impossible: (1) if a plant is mutated via ODM and would be stated as GMO (according the legal opinions of Spanger and Kraemer) and the same mutation would be corrected also via ODM, this organisms would be biological identical to the parental one but would also be treated as GMO. (2) If a manufacturer alters a plant by introducing a point mutation and would apply for authorization (as would be required according to the legal opinion of Krämer and Spanger. Under European law, under Article 13(2) and Annex III B, Section D., No. 12) to place it on the market, they would be required to provide information how to detect and identify the GMO along with the application. This would be impossible for (1) and (2). Therefore, the BVL states: “In reality, however, this alleged GMO would not be distinguishable from a plant which had acquired the same point mutation naturally or by means of chemical- or radiation-induced mutagenesis …. Therefore, such a GMO would not be distinguishable from organisms which do not fall under the scope of the Directive. Ultimately, this means that it would be impossible to monitor, and its placing on the market would not be eligible for approval, because the application dossier is incomplete. This outcome cannot have been the intention of the legislator. Apart from that, this would mean that the EU’s so-called zero-tolerance rule for non-approved GMOs in seed could no longer be fully implemented. This, too, cannot have been the intention of the legislator.”

The legal analyses on behalf of the NGOs and BFN result in the conclusion that the organisms created using the ODM technique have to be considered as GMO, since the introduction of oligomers is a genetic technique and targeted mutagenesis is not a naturally occurring process. Krämer mentions: “…the context may be relevant for defining whether usage of oligonucleotides is regarded as a mutagenesis or genome editing/genetic engineering. The difference in the terms is due to the technical details of the technology. In summary, the process used in oligonucleotide technology makes use of genetic material prepared outside the cell and thus has strong parallels to genetic engineering. … With regard to Directive 2001/18, these differences are relevant. First, the Directive emphasises that the process is decisive for defining what is covered by the Directive and what is exempted. Second, if the process is regarded as a mutagenesis, the use of recombinant DNA might be used as the most relevant criterion; if the use of oligonucleotides is not regarded as mutagenesis, other criteria will also have to be taken into account. Third, mutagenesis is known for many years, while genome editing is a recent technology.” And Spanger states: “The fact that mutations as such [sic!] do occur naturally is of no importance in this context. Crucial for this assumption is the fact that the ‘not-natural appearance’ has to be assessed in an individual-concrete but not in a general-abstract way. The modifications caused by ODM and similar new techniques are carried out purposefully and lead to the incorporation into a host organism in which the nucleic acid molecules with certainty do not occur naturally. As this represents a target-oriented point mutation, Annex I A Part 1 No. 1 has to be applied to the relevant genome editing techniques.”

## Urgency for clarifying the legal status of GE in the EU

Many public researchers are seriously concerned about the situation in the EU regarding the current legislation for field release and cultivation of genetically modified plants (Directive 2001/EC/18; Directive 2015/412) and the lack of certainty for novel GE techniques in relation to existing Directives. To give but a few examples; public researchers from Sweden and the Netherlands recently expressed hesitation at using valuable and versatile GE techniques in their world-class fundamental and applied research, not knowing whether or not they would be allowed to carry out field trials with the resulting plants. Perhaps worse yet is the situation where research funding applications are being rejected solely for this reason of GE uncertainty (Abbott 2015), or when companies decide to move R&D investments out of the EU because of a similar reason.

As described above, several signals have already been delivered from some of the EU member states and from EFSA regarding the regulation of GE techniques. Another precedential case occurred in 2014 when two public research groups in Sweden independently asked the Swedish Board of Agriculture (SBA) whether they needed to apply for permission to carry out field trials with Arabidopsis plants mutated with the CRISPR/Cas9 method. The question was followed up in the spring 2015, and on 13 November 2015, SBA announced that the application of CRISPR/Cas9 induced mutations in plants should be considered as equivalent to mutagenesis and provided that no foreign DNA is left in the mutated plant and, therefore, excluded from Directive 2001/18/EC together with the techniques listed in Annex 1B. SBA´s interpretation suggested that the original intention to exclude mutagenesis from Directive 2001/18/EC was that the resulting mutations should be in focus and not the methods yielding these mutations. Otherwise, it may jeopardize any future method development (SBA 2015a, b). As explained earlier, the Competent Authorities of six EU member states have also announced that the ODM is not to be regulated as technology resulting in a GMO under Directive 2001/18/EC. All of these statements represent strong signals to the EC to not further delay resolving current legislative uncertainties for exploring GE techniques as well as to allow strict scientific reasoning to play a prominent role in risk assessment and regulation of these techniques.

Reacting to the concerns of public researchers in the EU regarding the possibility to use GE techniques in their research, the EPSO, representing more than 220 European public research institutes, delivered a statement in February 2015 welcoming the outcome of the NTWG report (2012) and calling on the EC to urgently provide a guideline document following the recommendations of this report and clarifying the legal status of NPBTs, including GE techniques. The EPSO statement emphasizes that “the legal definition of a GMO does not apply to most of the NPBTs and that these techniques either fall under the exemptions already established by the legislation (Authors’ comment: see Directive 2001/18/EC; Annex 1A part 2, Annex 1B) or should be exempted, as they do not differ from plants obtained by traditional breeding” (EPSO 2015).

The statement was updated and reiterated to the EC in December 2015, clarifying that the interpretation of the EU GMO legislation is both process- and product-based and arguing that this would help clarifying the legal status of NPBTs (EPSO 2015). Certain GE techniques would yield very different products, such as plants with point mutations rather than gene insertions, compared to those which are classified as GMO—and not exempted—in Directive 2001/18/EC, and they should, therefore, clearly not be regulated as GMO in the current legislation. We agree with this and would like to once again highlight the point that the EU GMO Directives are commonly misinterpreted, by proponents and opponents alike, as being strictly based on process and not product. However, as explained above, this is not the case. A similar view to ours has also been put forward by, apart from EPSO, several other prominent European biotech and science organisations, such as the EASAC (2015), the European Seed Association (ESA 2012) and the European Technology Platform ‘Plants for the Future’ (Plant ETP 2012), as well as the Advisory Committee of Releases into the Environment (ACRE 2012), the Biotechnology and Biological Sciences Research Council (BBSRC 2014) in the UK and the BVL in Germany (BVL 2015d). On the other hand, Kuzma (2016) recently claimed that it is necessary to avoid the polarisation of process- vs. product-based interpretations to move forward in the debate, arguing that this regulatory dichotomy is neither logical nor scientific. Whereas we agree that any future system needs to be non-discriminatory in every aspect, we still want to point out that it is necessary to look at the resulting product to make sense of today´s legislation in the EU. If not, then the issue of implementing traceability measures would become impossible for certain products developed by GE techniques, i.e., they would be technically impossible to differentiate from products developed by classical (unregulated) mutagenesis, rendering the legislative procedure meaningless.

## Situation in the US, Canada and Argentina

As pointed out, the regulation of biotechnology in the EU dealing with GMOs is in principle of both process- and product-oriented, but their interpretation is in practice predominantly focused on the production process and if this process is leading to a GMO or not. In contrast to this, in the US, the assessment of the risk posed by the resulting organisms to human beings, animals or the environment is predominantly based on the end product and not the technological process (NRC 1989). A strong precautionary principle is implemented in the US law concerning biotechnology but nevertheless the process during which a GMO is produced is not considered to be dangerous per se and neither is the transfer of genetic material between organisms according to the US law. In Canada, a different regulation concerning plants, called “plants with novel traits,” is present. This regulation is based on the Plant Protection Act from 1990 and solely considering the novel trait of a plant, regardless which technology was used to produce it (e.g., biotechnology, conventional breeding or mutagenesis) (The Plant Protection Act 1990).

The regulatory system working in the US has been developed over the last five decades, and already in 1984, a Coordinated Framework for the Regulation of Biotechnology was issued (Lynch and Vogel 2001). This document is still the key document on biotechnology in the USA. Three agencies, such as EPA (Environment Protection Agency), USDA (US Department of Agriculture) and FDA (Food and Drug Agency), became responsible for regulating biotechnology, including genetic engineering. The FDA is responsible for medical products derived from biotechnology, the USDA for transgenic plants and the EPA for pesticidal plants and genetically engineered microbial pesticides (e.g., Bt-toxin). Concerning transgenic plants, a fast growing number of events have been deregulated by the USDA, which can be found in the APHIS database (Animal and Plant Health Inspection Service). Starting in 1992, a total number of 121 events have to date undergone deregulation by the USDA, including 19 different plant species, such as apple, corn, cotton, potato, tomato and others. The agencies involved based their decision rather on the scientific data which have been collected in the frame of risk assessments with respect to possible risks for humans, animals or the environment than on the biotechnological process which produced the new trait. Therefore, in view of the US regulatory system, genome editing by ODM or SDN should not be a matter of regulation as long as no pest sequences are integrated in the plant genome. The pure editing process involving only base mutations should not be regulated, because it does not pose a new risk to humans or the environment as long as it does not code for any pest sequence. Sustaining this view, the USDA stated already in 2004 that ODM is comparable to mutagenesis and will be most likely not in the focus of regulation in the US (Wolt et al. 2015). The first example for approval of genome editing in plants which is still in the launch phase for the US is the canola event 5715 (Cibus Inc., San Diego). To handle GE and further new biotechnology developments, a memorandum was passed in 2015 in the US which claims for a modernization of the US regulatory system (Memorandum 2015). In this text beside other aspects, it is clearly pointed out that future biotechnology regulation in the US should be based on the best available science; it should be transparent and efficient and should promote public confidence in the oversight of the products. These goals will be reached by the establishment of a Biotechnology Working Group as part of the Emerging Technologies Interagency Policy Coordination Group (ETIPC). The group will consist out of members from the Executive Office, FDA, EPA and USDA and will be coordinated with other Federal agencies (Memorandum 2015).

A Canadian system for GMO regulation is in principle not existent, as there is no specific regulation for the evaluation of a specific production process. The decision if, for example, a new plant is dangerous for humans or the environment is solely based on its novel inherent trait (The Plant Protection Act 1990). The definition of a novel trait is given by the Canadian Food Inspection Agency: “A plant with a novel trait (PNT) is a plant that contains a trait which is both new to the Canadian environment and has the potential to affect the specific use and safety of the plant with respect to the environment and human health. These traits can be introduced using biotechnology, mutagenesis, or conventional breeding techniques.” (Canadian Food Inspection Agency 2015a). This is a very pragmatic view which can lead to the situation that plant varieties harboring a novel trait have to be evaluated for potential risks even if conventional breeding or mutagenesis was used. For instance, in the case of crop herbicide resistance phenotypes, these have been developed by conventional breeding, mutagenesis and transgenesis as well as genome editing and subsequently evaluated and approved by Canadian regulators (Canadian Food Inspection Agency 2015b; Wolt et al. 2015). The Plant Protection Act also covers the invasive potential of new plants, and one of its main objectives is to prevent import, export or spreading of pests in Canada. In principle, this way of legislation is the logical result of the precautionary concept, but in view of a long history of safe use of such plants, it is not ideal for breeders which are developing new varieties by conventional breeding processes like introgression breeding. In 2016, 100 different plant events involving biotechnical modifications have been approved by the CIFA (Canadian Food Inspection Agency) which are recorded as decision documents in the Guidance Document Repository (GDR) (http://www.inspection.gc.ca/plants/plants-with-novel-traits/approved-under-review/decision-documents/eng/1303704378026/1303704484236). Concerning the genome editing technology, the Canadian government already approved the above-mentioned canola plants generated using the ODM technology (Canola Event 5715, Cibus Inc., San Diego) and most likely will approve new plants made by the use of genome editing techniques, such as CRISPR/Cas9 or others, summarized as SDN techniques.

In view of the different regulation processes performed in different countries, GE is, on one hand, a new kind of biotechnology, but, on the other hand, highly predictable when it comes to the risks which can occur using the technology. The principal intrinsic risks of the GE process are the same as in conventional mutagenesis (unintended base mutations off-side the target locus often called off-target activity) but to a much lesser extent, as the side mutations of GE are lower by orders of magnitude (EFSA 2012).

Argentina is the third largest grower of genetically modified crops in the world by 2014 (GLRC 2014). The use of GMOs in agriculture and food is regulated by the general Law on Seeds and Phytogenetic Creations (LS) and by the Law on the Promotion of the Development and Production of Modern Biotechnology (LB). The LS is covering all issues affecting the commercialization of crops and their import or export. In respect to genetically modified seeds, an additional registration in the National Registry of Operators of Genetically Modified Plant Organisms is mandatory (Resolucion 46/2004 2004). The LB is covering the legal issues connected to the promotion of modern biotechnology in Argentina, including research and production projects. The responsible authority for release and commercialization of GMOs is the Secretary of Agriculture, Livestock, Fisheries and Food. The granting of a permit to release or commercialize a GMO in Argentina is done case by case and depending on its assessments in regard to biosafety standards, food safety standards and additionally on an evaluation of the impact a commercialized GMO will have on Argentina´s trade. This system is—as the US and Canadian—focused on the evaluation of the new trait of a given GMO than on its production process per se. In contrast to the EU where only an assessment of potential risks has to be performed, an additional risk/benefit analysis can influence the approval process in Argentina. As of May 2015, Argentina became the first country to make its resolution on the regulatory status of NPBTs publicly available. The resolution determines that all crops derived through the use of NPBTs, and thus modern biotechnology, are to be reviewed on a case-by-case basis. However, the definition of a GMO is still missing approval, and real-world cases will have to show the practicalities of the resolution (Schuttelaar and Partners 2015).

The authors welcome the Argentinean practice to offer researchers and applicants the possibility to discuss future risk assessment and regulation of plants produced by means of NPBTs with the regulators. In a case-by-case approach, it will be evaluated in advance if the particular plant will fall under the scope of the Argentinean GMO regulation or not. This valuable procedure will save an enormous waste of time and effort for both developers/researchers and regulators (Whelan and Lema 2015) and will provide the legal certainty which is missed in Europe to date.

## Conclusions and perspectives

The legislation put in place to regulate the development and commercialization of GMOs in the EU was originally intended to handle issues of uncertainty and safety. Scientific progress has since provided us with a wealth of knowledge about the genetic structure of, inter-relationship between, and exchange of genetic material between a multitude of organisms from all major kingdoms, such as plants, animals, fungi and bacteria. In the context of safety issues, plants modified through GM or GE techniques should, therefore, be discussed in relation to the “natural baseline” of genetic variation that exists in nature. In nature, genetic alterations occur all the time, such as nucleotide sequence changes, intragenomic rearrangements of DNA, and the acquisition of foreign DNA segments by horizontal gene transfer. Within species, genetic variation, including SNPs and retrotransposons, can be enormous, and horizontal gene transfer is probably much more commonplace than we previously thought (Jansson 2015; Kyndt et al. 2015). This is all part of natural biological evolution. Similar genetic alterations may take place with GM as well as novel GE techniques. Estimations or assumptions of risk should, therefore, be of the same order of magnitude as for those changes involved in natural genetic variation, or for that matter, also in the conventional breeding methods (Arber 2010). From a scientific point of view, it is, therefore, reasonable to assume that genetic alterations caused by currently available GE techniques do not per se pose a “higher-than-natural” risk.

The original intention of the legislators was certainly also to include the aspects of both process and product in the definition of a GMO; hence, Directive 2001/18/EC defines a GMO as an organism “in which the genetic material has been altered in a way that does not occur naturally by mating and/or natural recombination”, and further describes GM techniques as “recombinant nucleic acid techniques involving the formation of new combinations of genetic material”. Since Directive 2001/18/EC includes, as a criterion, the formation of new combinations of genetic material, the product itself also has to be scrutinized whenever a particular technique is put in relation to the GMO regulation in the EU. This is also in compliance with the definition of Living Modified Organism (LMO) established in the Cartagena Protocol on Biosafety. The one-sided focus on a process-oriented interpretation may in fact be erroneous and misleading. When the argument is put forward that GE techniques should automatically be classified as genetic engineering and regulated accordingly, simply based on the fact that the steps of recombinant nucleic acid techniques are involved, it is commonly overlooked that the current legislation is based both on process and product (Abbott 2015). This erroneous argument becomes even more problematic when it is considered that the products of certain GE techniques are in several cases indistinguishable from those developed by conventional or mutation breeding. In our opinion, in the case, the use of a certain technique has not resulted in the incorporation of foreign DNA or any novel genetic combination, then the resulting plant cannot be regulated as a GMO according to the intention or the definitions of Directive 2001/18/EC. As explained previously, this view has also been expressed by the Competent Authorities in several EU member states as well as by EFSA, encouraging the EC to take this under consideration in the preparations for a legal interpretation for how novel GE techniques may be regulated according to the current EU legislation. Failure to adapt the regulatory system to fully be able to utilize the novel GE techniques may have—and is already having—severe negative impact on research and innovation in the EU.

Other regulatory systems have been proposed lately. Huang et al. (2016) suggested a five-step procedure for GE crops including the following: (1) minimizing the risk of unintentional release from laboratories and field trials, (2) demonstrating the absence of foreign DNA sequences, (3) documenting DNA changes at the target site, (4) ensuring the absence of unintended secondary editing events, and (5) including the documentation of the above four points in the application. If all five steps are satisfactorily met, the GE crops should be subject only to the same rules that apply to conventionally bred cultivars before commercial release. Miller (2010) and Barton et al. (1997) presented the “Stanford Model” for regulation of field trials with GM/GE plants, being unsatisfied with the lack of proportion between risk and regulatory scrutiny. This model stratifies organisms according to risk in field trials, and is analogous to existing regulatory regimes, such as those for quarantine regulations for plants or animal pests, and also to the US government’s approach to handling dangerous pathogens or other microorganisms in the laboratory. The advantage of the Stanford Model is that it is sufficiently flexible to accommodate differences in regulatory authorities’ preferences for greater or lesser regulatory stringency, as long as the risk factor of each category is coupled with an appropriate and relative regulatory requirement. Another similarly flexible model has been presented by Araki and Ishii (2015), setting up the continuum of genetic alterations in a range from minor (leaky or null mutations) to major (transgenesis) changes. Four levels of stringency in the regulation are imposed along this range, allowing policy makers to shift towards more permissive regulation for certain genetic alterations as evidence of safety accumulate. This model would also promote global harmonisation of regulatory frameworks. The benefit of this model is, in our opinion, that it has the flexibility to start off with a relatively process-directed system of regulation, that is politically acceptable to many stakeholders today while allowing a shift towards more product-based interpretations as scientific evidence accumulate and the products gain a safe history of use.

All of these models have their virtues and should serve as inspiration for the development of a more dynamic regulatory system in the EU that is flexible enough to accommodate any novel plant research and breeding techniques.

### **Author contribution statement**

All authors wrote the manuscript. All authors read and approved the manuscript. TS prepared the figures.

## Box 1

### Zinc finger nuclease (ZFN) techniques

The authors suggest to replace the term by Site-Directed Nuclease (SDN) techniques in accordance with recent developments and to the EFSA scientific opinion addressing the safety assessment of plants developed using Zinc Finger Nuclease 3 and other Site-Directed Nucleases with similar functions (EFSA 2012). SDN are customized molecules consisting of a DNA-recognizing domain (protein or RNA) and a nuclease (protein) that cuts (resulting in double-stranded break; DSB) or nicks (resulting in single-strand break) double-stranded DNA. SDN allow site-specific mutations (SDN-1) in the genomes or the site-specific integration of DNA fragments (SDN-2, SDN-3). Genes coding for SDN can either be stably integrated into the genome (in this case, the offspring is still carrying the transgenic SDN and have to be selected for the loss of the transgene to not constitute a GMO according to the definition of Directive 2001/18/EC) or SDN can be expressed transiently, i.e., from a plasmid vector to generate the desired change in the genome. In the latter case, the mutations will be stably inherited even after vector degradation. The third possibility is a vector-free mutation system in which SDN are directly integrated as proteins and/or an RNA/protein-complex into the cells; in this case, the SDN will be quickly degraded by the cell, but the mutation stays stably inherited.

### Site-directed nucleases I (SDN-1)

SDN are delivered to the cells without repair template. The SDN generates site-specific breaks which are further repaired by the cellular repair mechanisms of the host, mainly by non-homologous end joining (NHEJ). This leads to site-specific mutations in one to only a few base pairs or to short insertions or deletions.

### Site-directed nucleases II (SDN-2)

SDN are delivered to the cells along with a repair template homologous to the targeted DNA site differing only in one to a few base pairs and spanning several base pairs to a few kilo bases. The SDN generate site-specific breaks (or nicks), which are repaired via the cellular repair mechanisms (NHEJ) or homologous recombination (HR), leading to changes from one to a few base pairs (>20 bp) through HR using the homologous repair template. Site-specific mutations may also occur (see SDN-1).

### Site-directed nucleases III (SDN-3)

SDN are delivered to the cells along with a piece of DNA which can be up to several kilo bases long, the ends of which are homologous to the DNA sequence flanking the designated site of break induction. The region between the homologous ends does not have to be homologous. The breaks are further repaired via the cellular repair mechanisms (HR or NHEJ). In the case of an HR repair, the DNA stretch can be inserted into the genome in a site-specific manner, leading to the insertion of larger pieces of DNA. Alternatively, site-specific mutations may also occur, derived from NHEJ (see SDN1).

### Zinc finger nucleases

ZFN are customized combinations of two protein domains, the zinc finger DNA-binding domain and an endonuclease domain (most frequently FokI). The α-helices in the zinc finger DNA-binding domain define the binding of three base pairs depending on the structure. In nature, there are >1000 known zinc finger domains. Typical ZFN consist of three to six zinc finger domains each recognizing 3 bp. All ZFN work as pairs, since FokI only introduces DSBs as a dimer. The targeted DNA sequence can vary between 18 and 36 bp in length.

### TALE nucleases

Transcription activator-like effector nucleases are a combination of an endonuclease (most frequently FokI) with a subset of DNA-binding protein domains derived from *Xanthomonas spec*. TALE nucleases consist of multiple 33–35 amino acids long repeats each binding to a certain single base pair. The specificity is defined by the amino acids nos. 12 and 13 in the repeats. TALEN are commonly used as pairs to introduce double-strand breaks (DSBs) to the DNA, as FokI only introduces DSBs as a dimer. The DNA sequence targeted by a TALEN pair is varying in most cases between 30 and 60 bp.

### CRISPR/Cas9 nucleases

Clustered regularly interspaced short palindromic repeat—associated endonucleases are a combination of the endonuclease Cas9 with a fusion of two RNA molecules. The RNA directs the nuclease to a targeted DNA sequence via base pairing. The DNA guidance is achieved by a designable guide RNA which has to be adjacent to a DNA motif which is specific to the chosen Cas9 nuclease (commonly NGG or NAG). The DNA targeted by a CRISPR/Cas9 nuclease is typically 16–20 bp long.

### Oligonucleotide-directed mutagenesis (ODM)

Oligonucleotide-directed mutagenesis can be used for the induction of targeted mutations of a single or a few adjacent nucleotides in the genome. Typically used oligonucleotides are single-stranded DNA or chimeric oligonucleotides consisting of mixed DNA and RNA bases. These oligonucleotides are chemically synthesized to share homology with the targeted DNA sequence but not with the nucleotides which are desired to be changed. The common explanation is that the cellular repair mechanisms recognize the mismatched pairing and induce their correction. The DNA targeted by ODM spans one to a few adjacent base pairs. The oligonucleotide used for this modification is between 20 and 100 bp long.

### RNA-dependent DNA methylation (RdDM)

RNA-dependent DNA methylation induces the transcriptional silencing of targeted genes via the methylation of the corresponding gene and/or promoter sequence. To obtain such methylation patterns genes, encoding RNAs with homology to the desired target are used. These genes give rise to double-stranded RNA which induces, after being processed by the cell, the methylation of the targeted genomic region. Since these methylation patterns are inheritable, the trait can be found in the following generation even after outcrossing of the transgene by segregation. RdDM is mainly used for gene silencing. The interfering RNAs are between 21 and 24 bases long.
